# A Decision Tree for Donor Human Milk: An Example Tool to Protect, Promote, and Support Breastfeeding

**DOI:** 10.3389/fped.2018.00324

**Published:** 2018-10-31

**Authors:** Shelley Brandstetter, Kimberly Mansen, Alessandra DeMarchis, Nga Nguyen Quyhn, Cyril Engmann, Kiersten Israel-Ballard

**Affiliations:** ^1^School of Nursing, University of Washington, Seattle, WA, United States; ^2^Maternal Newborn Health and Nutrition, PATH, Seattle, WA, United States; ^3^Department of Global Health, University of Washington, Seattle, WA, United States; ^4^Seattle Children's Hospital, Seattle, WA, United States; ^5^PATH Vietnam, Hanoi, Vietnam; ^6^Department of Pediatrics, University of Washington, Seattle, WA, United States

**Keywords:** breastfeeding, decision tree, donor human milk, pre-term, NICU

## Abstract

Despite decades of breastfeeding promotion, exclusive breastfeeding rates for the first 6 months of life remain low: around 40% globally. Infants that are admitted to a neonatal ward are even less likely to be exclusively breastfed. Lactogenesis is frequently delayed in mothers that deliver early, with the added burden of separation of the unstable newborn and mother. For such vulnerable infants, donor human milk is recommended by the World Health Organization, UNICEF, and professional organizations as the next best alternative when mother's own milk is unavailable and can serve as a bridge to full feeding with mother's own milk. Hospital support of optimal breastfeeding practices is essential with thoughtful integration of donor human milk policies for those infants that need it most. We propose a decision tree for neonatal wards that are considering the use of donor human milk to ensure donor human milk is used to replace formula, not to replace mothers' own milk. By first evaluating barriers to full feeding with mother's own milk, healthcare workers are encouraged to systematically consider the appropriateness of donor human milk. This tool also seeks to prevent overuse of donor human milk, which has the potential to undermine successful lactation development. In settings where donor human milk supplies are limited, prioritization of infants by medical status is also needed. Readily available and easy-to-use tools are needed to support healthcare staff and mothers in order to improve lactation development and neonatal nutrition.

## Introduction

Breastfeeding is well known as the optimal source of nutrition for infants, and is recommended as the sole food until 6 months of age (1–3). Although this public health message has been widely disseminated, exclusive breastfeeding rates for the first 6 months are not optimal, and are estimated to be <40% globally (4). Premature, low-birth-weight, and small-for-gestational-age babies are at increased risk for feeding issues. Concurrently, mothers of premature infants are also at risk of delayed lactogenesis, impacting short-term ability to express or pump sufficient volumes to meet the infant's immediate needs and increases the potential for insufficient long-term breast milk supply (5–7). Prematurity of the infant is associated with an underdeveloped suck, swallow, and breathe reflex: necessary for safe and effective oral feeding (8). The combination of these factors demands increased attention for this vulnerable mother-infant dyad to receive additional support for lactation and neonatal nutrition.

Being born too soon or too small is a nutritional emergency, requiring close monitoring to ensure adequate growth (9). The increased nutritional needs of the small baby require thoughtful, evidence-based facility policies to ensure all infants have access to human milk to reach their growth goals. Compared to infants that receive exclusive human milk diets, infants that receive formula are at increased risk for complications such as necrotizing enterocolitis, bronchopulmonary dysplasia, and sepsis (10–13). For mothers who experience delayed lactogenesis, optimal alternatives are needed to ensure optimal infant health and to protect the mother's ability to build her milk supply.

## Background for the development of this decision tree

The World Health Organization, UNICEF, and other policy leaders recommend donor human milk (DHM) as the preferred alternative if mother's own milk is not available (2, 14–17). Preterm formula is preferred to term formula for premature infants where DHM is unavailable (18). Donor human milk is expressed breast milk donated by one mother, then processed by a human milk bank to be given to another mother's infant. Guidelines for the prioritization of donor human milk vary by setting, with no global unified guidance for the use or prioritization of donor human milk. Although the minutiae of prioritization criteria must be decided at the national or local level based on a number of factors including supply, broad prioritization guidance is needed to prevent the misuse, including overuse, of DHM, at the expense of providing the optimal nutrition of mother's own milk.

One benefit of DHM is its ability to serve as a bridge to full feeding with mother's own milk (19, 20). In addition to the stresses of premature birth, the mother may also experience challenges with lactogenesis (5, 21). DHM can provide the neonate with a more optimal source of nutrition than formula, while the mother builds up her breast milk supply through alternative methods of expression. Lactation support is vital in the first few days after birth, especially for mothers of premature infants (5). The volume of feeds required by premature infants are minimal, Even a small amount of DHM per infant could provide enough volume to allow the mother the time to come-to-volume, resulting in her own milk being available for her infant (22).

Evidence suggests there may be potential overuse of DHM, as it may be seen as a more convenient source of nutrition than expressed mother's milk for the hospitalized infant (21). Settings may face a variety of barriers to optimal feeding with mother's own milk, including transportation challenges, harried health care workers, and fractured health care systems. In areas that are not set up to support mothers, routinely separate mothers and infants, and are not baby-friendly, DHM may truly be more easily *available* than mother's own milk, but should not be considered an equal replacement. Systems are needed that support mothers to develop their own milk supply and prioritize their milk for their infants. Research has shown that mothers who reach full volume by 14 days post-delivery, estimated as 500 mL of breast milk per day, are more likely to be able to continue breastfeeding at discharge (22). Robust hospital policies and guidelines are needed to ensure all efforts have been made to prioritize mother's own milk and support maternal lactation, not only prior to the allocation of DHM, but routinely during the infant's hospitalization.

In order to support breastfeeding, ensure optimal nutrition for all infants, and prevent misuse, a decision tree was requested by clinicians and experts in human milk banking and neonatal care. This decision tree was intended to help guide prioritization and allocation of DHM, as well as evaluate barriers to feeding with mother's own milk.

## Overview of the decision tree

**Figure 1:**
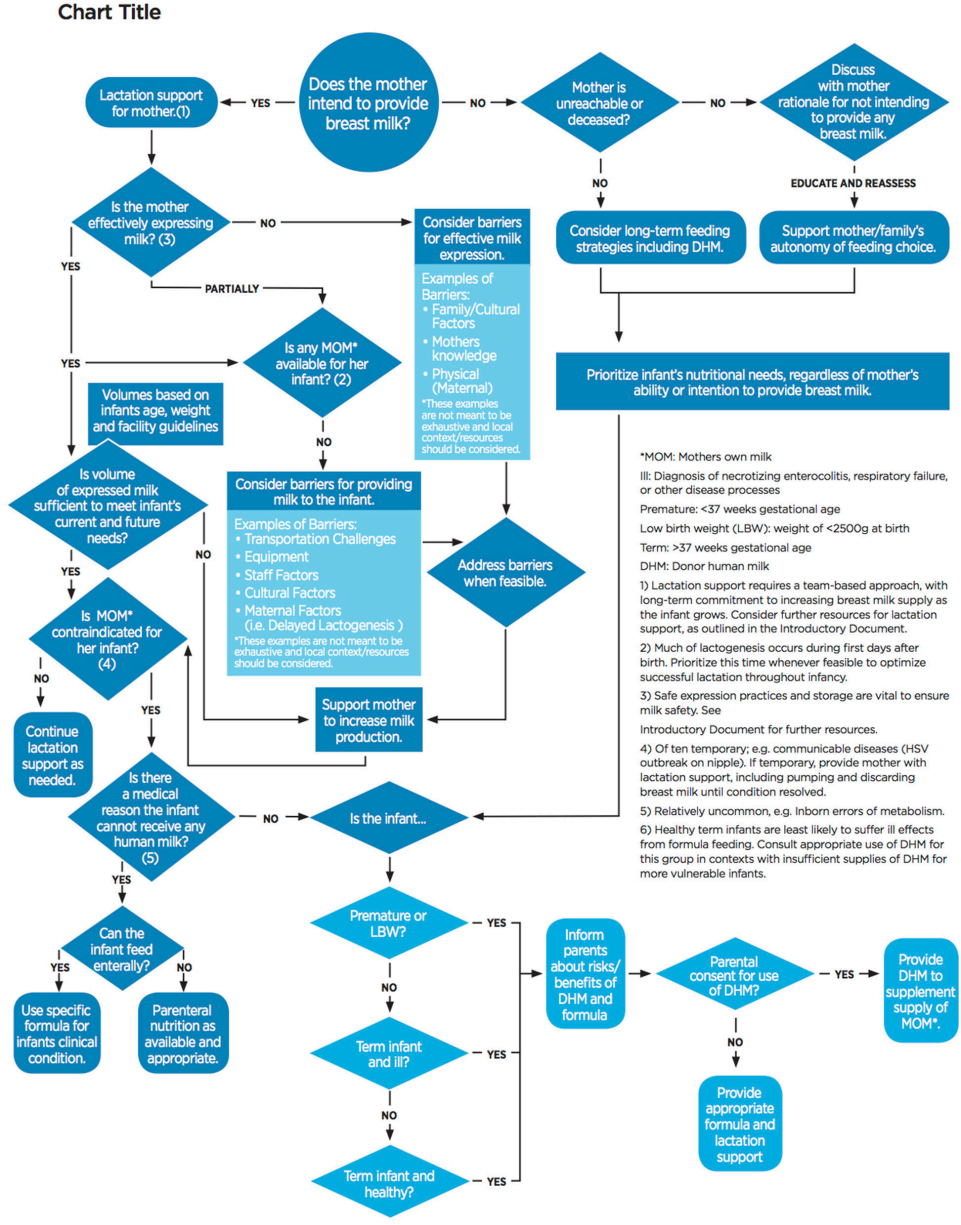
Decision Tree.

The overarching goal of the decision tree is to first protect, promote, and support breastfeeding, by encouraging thoughtful use of DHM, taking into account neonatal needs and maternal lactation considerations. This decision tree (Figure 1), when utilized with early and essential newborn care as an integrated package of interventions, including kangaroo mother care, will help ensure optimal neonatal nutrition and maternal lactation support.

A decision tree model was chosen to provide clinicians with a visual guide that evaluates and promotes maternal lactation support. This should occur at neonatal admission and whenever nutrition orders are being reevaluated throughout the hospital stay. The staff member should first evaluate if the mother intends to provide breast milk to her infant. If the mother does not plan to provide breast milk, staff are encouraged to explore barriers to breastfeeding or expressing breast milk, and ensure that her decision is informed. In cases where the mother is physiologically unable to provide any breast milk (a rare event), or deceased, additional counseling, or education is not appropriate or feasible. For all other mothers, education and assistance should be provided; if the mother is still unwilling to provide breast milk to her infant, staff must respect her autonomy to feed her infant how she chooses. Regardless of the mother's ability or willingness to breastfeed, the source of nutrition should be determined based on the needs of the infant; in some cases, DHM may still be appropriate.

In situations where the mother is willing and able to provide her breast milk to her infant, but sufficient volumes are not available for her infant's feed, the decision tree will assist the health care worker in evaluating next steps. In settings without a mechanism for the mother's expressed breast milk to be available in the neonatal ward, the decision tree will prompt clinicians to evaluate these barriers. For example, a mother delivers prematurely due to her poor health. She is remains hospitalized in the maternity ward while the infant is hospitalized in the neonatal ward. In health systems without strong communication structures between different units or hospitals, the ill mother may be pumping and freezing her breast milk, while her infant is receiving DHM or formula due to lack of maternity-neonatal staff communication and ability to efficiently transfer milk across units. Using this decision tree will prompt staff in these scenarios to discover and address these barriers.

When infant needs are unmet despite best-efforts to feed with mother's own milk, only then are staffs encouraged to use DHM. In settings where supply of DHM is limited, these small babies are prioritized by medical conditions: those that are at highest risk for morbidity and mortality should receive DHM first. This determination was made based on available evidence for use of DHM for efficacy and cost-effectiveness (23–26). Need for DHM should be reassessed routinely by clinicians to ensure ongoing need. Ideally, infants will transition to full feeding with mother's own milk during hospitalization. Early, and ongoing lactation support and encouragement to build up a mother's milk supply is vital if mothers are to successfully breastfeeding their infants post-discharge.

## Preliminary usage and future steps

A preliminary version of the tool was pre-tested in neonatal wards in India and Vietnam. Clinical staff in India recommended simplification of language used, and target use by new nursing staff and resident physicians. As the foundational elements of this tool are already integrated into current practice, the decision tree was considering most applicable for staff education and training. Also identified was the opportunity to modify facets of this into breastfeeding promotion education adverts for parents. Assessment of the decision tree in Vietnam provided feedback that the tool can be adapted into other formats, such as a decision checklist used by staff on admission to the NICU. This provides further evidence that the decision tree may have uses beyond its original intent.

Future steps for the decision tree include rigorous pre-testing and trialing the preliminary tool in additional settings to assess applicability, appropriateness, usability, and possible impact on increased use of mother's own milk, while also preventing overuse of DHM. This feedback will be integrated into the final decision tree, which will be globally accessible as part of a comprehensive package of resources for strengthening newborn nutrition. As differing settings will have specific policies and procedures that may not be covered by this decision tree, we will encourage adaptation of the tool to local settings.

## Conclusion

This decision tree fills a gap for systematically identifying and addressing barriers for vulnerable neonates to receive human milk. Although many hospitals have policies and protocols for the utilization and prioritization of DHM, these are not always readily available, nor do they address barriers to breastfeeding, provision of mother's own expressed milk, or neonatal nutritional needs. The intent for publishing this decision tree is to reframe the use of DHM to focus on provision of mother's own milk whenever possible. Hospitals and other settings that use DHM should evaluate their current systems and consider strategies for optimizing mechanisms to document and track current infant feeding practices. Better monitoring of actual infant feeding practices will facilitate improved accountability for prioritization of mothers' own milk.

Further research is needed to understand barriers and facilitators for optimal provision of human milk in the NICU in different settings globally. Current research supports the use of donor human milk for preterm and low-birth-weight infants, however evidence is limited in infants with other health conditions or beyond the first weeks of life. Future research may support the use in broader disease states, such as those infants with congenital heart defects, or those who are several months old. Additionally, enhanced indicators should be established to track global progress toward supporting mothers and use of human milk in NICU settings, beyond the traditional early initiation of breastfeeding indicators. Improved and rigorous data collection will help improve quality within the unit to ensure that all infants have the best start in life through equitable access to human milk.

## Author contributions

SB, KI-B, KM, AD, NN, and CE conceptualized and developed the decision tree. SB wrote the first draft of the manuscript. SB, KI-B, KM, and CE reviewed and finalized manuscript. All authors approved the final manuscript.

### Conflict of interest statement

The authors declare that the research was conducted in the absence of any commercial or financial relationships that could be construed as a potential conflict of interest.
